# 

**Published:** 1875-09

**Authors:** 


### Table of Contents.

### ORIGINAL COMMUNICATIONS.

Observations on the Influence of Consanguinity as the Medium of Propagating Typhoid Fever. *By T. S. Hopkins, M. D., Thomasville, Georgia* .......... 321

Cases of Intestinal Obstruction, from (probably) Malarial Causes, with some Reflections as to a probable cause of Intussusception. *By W. D. Hoyt, M.D., Rome, Georgia* .......... 329

Notes from Private Practice. *By J. G. Thomas, M.D., Savannah, Ga.* 335

Chloroform in Obstetrical Practice. *By Daniel Barry, M.D., Flatwoods, Tennessee* .......... 340

### OUR EXCHANGES.

A Review of the Treatment of the Principal Diseases at the German Hospital, Philadelphia .......... 343

The Cheaper Cinchona Alkaloids .......... 356

Denidation .......... 358

A Combination of the Cutaneous and Musculo-Cutaneous Methods of Amputation .......... 359

Mammary Abscess .......... 360

Asthmatic Mixture .......... 361

Fascinations of Science .......... 362

A New Adhesive Plaster. .......... 362

Sir Thomas Watson on the Treatment of Habitual Drunkards .......... 364

Medical Legislation in Tennessee—Liquor Sennæ .......... 365

Dr. Geo. Sutton on Trichinosis—Cheap Office Sign .......... 366

Chloral Hydrate—The Dangers of Cantharides—Solution of Iodo-Bromide Calcium Compound. .......... 367

Charcot on the the Relief of Hysterical Seizures by Compression of the Ovaries—Fees in New York City. .......... 368

Confessions of a Quack Doctor .......... 369

The Law of Abortion .......... 370

Prayer as a Medicine .......... 371

Experimental Science in Modern Education .......... 372

Testing Toughened Glass .......... 373

Recovery from Lightning Stroke—External Use of Chloral Hydrate .... 374

### BIBLIOGRAPHICAL.

A Report on the Hygiene of the United States Army .......... 375

Bulkley's Management of Eczema .......... 376

Michel's Anatomy of the Bullet Track and Cancroid of the Lower-lip—Ott on Physiological Action of Gelsemia—Sutton on Trichinosis—Steigner's Periodicals—Catalogues .......... 377

### EDITORIAL.

Advertising .......... 377

Warm Springs .......... 378

Removal—Membranous Dysmenorrhœa .......... 379

State Board of Health .......... 381

Crime of "Burking" .......... 382

State Board of Health—Dr. John M. Johnson .......... 384

